# Case Report: Coexpression of somatic *EGFR*, *BRCA1*, and *BRCA2* mutations in metastatic non-small cell lung cancer

**DOI:** 10.3389/fonc.2026.1964203

**Published:** 2026-10-08

**Authors:** Aaron Negin, Kush Sachdeva, Jared Weiss

**Affiliations:** 1 Southern Oncology Hematology Associates, Inspira Health System, Vineland, NJ, United States; 2 Division of Oncology, University of North Carolina School of Medicine, Chapel Hill, NC, United States

**Keywords:** adenocarcinoma, *BRCA1*, *BRCA2*, case report, *EGFR*, non-small cell lung cancer (NSCLC)

## Abstract

**Background:**

Treatment of non-small cell lung cancer (NSCLC) has evolved dramatically over the past decade with the advent of widespread genetic testing, immunotherapy, and targeted therapies. Treatments directed against epidermal growth factor receptor (EGFR) and other driver mutations have reduced toxicity and improved efficacy for selected patients.

**Case presentation:**

We present a 59-year-old man with metastatic NSCLC harboring coexisting *EGFR*, *BRCA1*, and RNA-binding motif protein 10 (*RBM10*) mutations and high programmed death-ligand 1 (PD-L1) expression. A *BRCA2* mutation was later detected on peripheral blood testing. The patient presented with a debilitating stroke that included dysphagia and hemiparesis. During his course of care, he received osimertinib, after which *EGFR* and *RBM10* variant allele frequencies (VAFs) increased on peripheral blood testing. He also received immunotherapy and olaparib. Despite high response rates reported in the literature, he did not respond to these agents. He had a response to chemotherapy but tolerated it poorly. Ultimately, given the lack of durable response and his inability to tolerate chemotherapy, the patient opted for palliative care.

**Discussion:**

Patients with actionable targets such as *EGFR*, *BRCA1/2*, or elevated PD-L1 generally have superior outcomes with targeted and combination therapy compared with patients who lack targetable options. However, coexisting mutations can interact and provide escape mechanisms. The lack of response in our patient is consistent with studies showing reduced efficacy of *EGFR*-directed therapy in the presence of *RBM10* loss-of-function (LOF) alterations. Further research in patients with multiple concurrent mutations is needed to improve treatment options for this group.

## 1 Introduction

Non-small cell lung cancer (NSCLC) treatment has evolved greatly over the past 20 years with the discovery of genetic and immunologic targets. The first targetable alteration identified was an epidermal growth factor receptor (EGFR) mutation in 2004 (1). EGFR tyrosine kinase inhibitors were subsequently moved into the first-line setting after the landmark IPASS trial demonstrated their superiority to chemotherapy in 2009 (2). Pharmaceutical research has further improved these therapies, yielding second-, third-, and even fourth-generation agents with greater efficacy and fewer adverse effects (3).

*BRCA1* and *BRCA2* mutations were first identified as risk factors for breast and ovarian cancer in 1990 and 1995, respectively (4). Eventually, these mutations were targeted therapeutically with poly(ADP-ribose) polymerase (PARP) inhibitors (5, 6). Indications were later expanded to other malignancies that share these mutations, including prostate and lung cancer (7).

Mutation testing remained slow and expensive in the early era of targeted therapy. Current commercial assays allow community physicians to test many patients for more than 600 gene mutations by next-generation sequencing (NGS) (8). Our understanding of cancer genetics expanded with the availability of testing, including finding multiple mutations in the same patient. These findings challenge the one mutation, one drug paradigm. Furthermore, testing can now be done serially in patients to understand the evolution of cancer genetics in a patient over time. In fact, molecular tumor boards have emerged in clinical care to handle this phenomenon (9, 10).

Here, we present a case of NSCLC with somatic *EGFR*, *BRCA1*, and *BRCA2* mutations that did not show a clinical response to targeted therapy.

## 2 Case presentation

A 59-year-old man of Chinese descent with no significant past medical history and a 40-pack-year smoking history presented to the hospital with sudden-onset focal neurologic deficits, including right-sided hemiparesis, dysphagia, and confusion. MRI of the brain demonstrated multiple acute embolic infarcts without intracranial metastases. Computed tomography (CT) showed multiple bilateral lung lesions and mediastinal lymphadenopathy without extrathoracic disease. Bronchoscopic biopsy revealed non-small cell lung cancer (adenocarcinoma). Tissue and liquid NGS panels (see **Table 1**) demonstrated a programmed death-ligand 1 (PD-L1) tumor proportion score (TPS) of 65%, an *EGFR exon* 19 deletion, and *BRCA1* loss-of-function (LOF). Of note, tissue NGS identified an *RBM10(LOF)* mutation that was not detected on contemporaneous peripheral-blood NGS.

**Table 1:** Pathology.

Pathology review	Results	Test
16 June 2025: tissue	PD-L1 22C3: TPS 65%	NeoGenomics, PanTracer NGS
	MSI: stable	
	TMB: 6.3 mut/Mb (low)	
	*EGFR* E746_T751del ([exon] 19)	
	*RBM10*(LOF)	
	Other mutations: *BAP1*, MAG12m	
1 July 2025: peripheral blood	*EGFR* E746_T751del ([exon] 19) (VAF 0.6%)	Tempus, xF Plus 523-gene
	*BRCA1* pQ491 (VAF 0.2%)	
	*BAP1* (VAF 1.3%)	
28 October 2025: peripheral blood	*EGFR* E746_T751del ([exon] 19) (VAF 3.1%)	Tempus, xF Plus 523-gene
	*BRCA1* pQ491 (VAF 0.9%)	
	*BRCA2* pQ1562 (VAF 0.9%)	
	*BAP1* (VAF 3.8%)	
	*RBM10* (VAF 3.7%)	
	*ZFHX3* (VAF 1.1%)	
8 July 26: germline genetics	No mutations, including pathogenic mutations, variants of unknown significance, or gross deletions/duplications.	Ambry Genetics, CancerNext-Expanded, 78 genes

The patient recovered partially from the stroke but continued to have significant weakness and malnutrition related to dysphagia and was therefore not considered a candidate for concurrent chemotherapy plus osimertinib, as studied in the FLAURA2 (First Line AURA) trial (11). He was started on single-agent osimertinib per the FLAURA trial (12). He tolerated the medication well but required multiple hospitalizations for dysphagia and seizures related to his stroke. Restaging scans at 2 months showed stable disease; however, imaging 3 months later demonstrated massive progression.

Repeat liquid NGS at that time showed that the *EGFR* mutation, while qualitatively unchanged, had increased in variant allele frequency (VAF) from 0.6% to 3.1%. An *RBM10(LOF)* mutation was now detected in blood at a VAF of 3.7%; this alteration had been identified on the original tissue assay but not on the initial liquid biopsy. An additional *BRCA2* mutation was newly detected in plasma at a low VAF of 0.9%. Given the *BRCA1/2* findings, germline testing was performed and revealed no pathogenic mutations.

Given the rapid progression, concerns about the limitations of targeted therapy, and high PD-L1 expression, the team discussed dose-reduced combination chemoimmunotherapy with the patient and family. After review of the risks, benefits, and alternatives, the team and family elected to initiate dose-reduced carboplatin, pemetrexed, and cemiplimab (13). The patient had a difficult course but completed four cycles, with a partial response on CT and marked clinical improvement. Chemotherapy was discontinued, and single-agent immunotherapy was continued. He tolerated maintenance immunotherapy well and maintained a good quality of life; however, imaging after four additional doses showed moderate progression.

The case was reviewed at tumor board. The patient was considered not to be a candidate for further chemotherapy, and therapy was switched to olaparib per the TAPUR (Targeted Agent and Profiling Utilization Registry) trial (14). He had difficulty tolerating the drug but was able to continue at a reduced dose. His cancer continued to progress on the first restaging evaluation. Given the lack of response to multiple lines of therapy (osimertinib, cemiplimab, and olaparib) and poor tolerance of chemotherapy, the patient and family opted for palliative care. The clinical course of the patient is summarized in **Table 2**.

**Table 2:** Clinical course.

Date	Clinical event	Comment
June 2025	Presented with stroke and found to have lung lesions. Concern for brain lesions as a cause of stroke, although not definitive.	Debilitated from stroke, including an inability to swallow and R hemiparesis.
June 2025	Bronchoscopic biopsy: adenocarcinoma of the lung.	NGS: EGFR (*exon 19 del*), *BRCA1*, *RBM10*, and multiple other nonactionable mutations. TMB-low. PD-L1 65% TPS.
July 2025	Osimertinib initiated.	Patient was too debilitated to receive concomitant chemotherapy.
August 2025	PEG tube placement due to inability to swallow with multiple admissions.	
September 2025	CT chest: slight progression.	Osimertinib continued.
December 2025	CT chest: massive progression.	Treatment change: carboplatin, pemetrexed, and cemiplimab
March 2026	CT chest: partial response.	Switched to single-agent cemiplimab after four cycles.
June 2026	CT chest: progression.	Switched to olaparib for 2.5 months.
August 2026	CT chest: progression.	Palliative care after discussion with family.

## 3 Discussion

Targeted therapy has become a mainstay of treatment for NSCLC. Patients with *EGFR* mutations have been studied across multiple generations of targeted agents. FLAURA demonstrated an overall response rate (ORR) of 77% in newly diagnosed metastatic NSCLC (11). Further clinical benefit was achieved by adding chemotherapy to these targeted agents (12).

In patients with metastatic breast cancer and *BRCA1/2* mutations, the PARP inhibitor olaparib produced an ORR of 60% (15). The TAPUR trial evaluated targeted agents approved for other malignancies for use in NSCLC. Among 25 patients with NSCLC harboring *BRCA1/2* mutations, the disease-control rate was 41% (response or stable disease lasting at least 16 weeks) in this rare patient population (14, 16).

Our patient had a somatic rather than germline mutation. A large meta-analysis in breast cancer found a nonsignificant trend toward lower responses in patients with somatic alterations (17). The numbers were small, however, and both cohorts derived benefit. PARP inhibitors are approved for other diseases with somatic *BRCA1/2* mutations, including prostate cancer. Our patient’s liquid-biopsy VAF was very low. Although this finding may represent a subclone, some studies have reported greater responses at low VAF (18). Given his poor performance status at the start of olaparib and the small TAPUR cohort, the significance of his lack of response is unclear.

The understanding of response to checkpoint immunotherapy continues to evolve. PD-1/PD-L1-directed agents appear more effective in patients with higher PD-L1 expression by immunohistochemistry (19). Additional studies have shown activity in tumors with microsatellite instability or high tumor mutational burden (TMB) (20, 21). KEYNOTE-789 and CheckMate 722 were large phase 3 trials of pembrolizumab and nivolumab, respectively, combined with chemotherapy in *EGFR*-mutant NSCLC. Both showed only marginal, statistically nonsignificant gains in response rate and progression-free survival (22–24).

Our patient did not respond to *EGFR*-targeted therapy with osimertinib. An exploratory analysis of FLAURA data examined co-mutations as sources of acquired resistance to *EGFR* inhibition (25). Review of his NGS identified a LOF mutation in RNA-binding motif protein 10 (RBM10). *RBM10(LOF)* coexists with EGFR mutations and confers resistance to EGFR inhibitors *in vitro*. Specifically, *RBM10(LOF)* reduces EGFR inhibitor-mediated apoptosis by shifting the balance toward antiapoptotic signaling (26). In a series of 50 patients with metastatic NSCLC and *RBM10* alterations, including 20 with coexisting *EGFR* mutations, clinical responses were poor (27).

After 5 months of osimertinib, the *EGFR* VAF rose from 0.6% to 3.1%, consistent with expansion of that clone. The *RBM10(LOF)* mutation, initially detected only in tissue, appeared in blood at a VAF of 3.7% after osimertinib. Consistent with published data, this pattern suggests that *RBM10(LOF)* may have served as an escape mechanism for the *EGFR*-mutant clone. Of note, while *EGFR* and *RBM10* rose during serial evaluation, we cannot prove they are part of the same clone.

While these tools have dramatically changed our understanding of the genetics of cancer, there are still significant limitations. Targeted short-read NGS does not comprehensively capture large structural variants or complex rearrangements and therefore provides a clinically useful but incomplete representation of tumor genomic architecture (28). Furthermore, low VAF findings in peripheral blood testing may reflect subclonal disease. The increases in the VAFs of EGFR and RBM10 in our patient’s serial testing may reflect this process.

Others have examined the impact of coexisting driver mutations. In a cohort of 5,420 patients with metastatic NSCLC from Halifax, Canada, 3,142 (58%) had a driver mutation, and 212 (4%) had coexisting drivers. Patients with coexisting mutations had significantly worse progression-free and overall survival than those with a single driver, regardless of which specific alterations were present (29). It remains unclear whether tumors with multiple drivers are biologically distinct or whether the additional drivers simply provide escape routes.

Widespread NGS and targeted therapy now allow molecularly individualized treatment of NSCLC. As this case illustrates, however, the older paradigm of one drug against one mutation has limitations. The evolving complexity of cancer genomics requires further study. Additional research is needed to understand and target coexisting mutations.

## 4 Patient perspective

The case report was composed after completion of therapy. The patient was informed about his unique case during his care and strongly endorsed this publication with the desire to help others. The family was supportive both during his care and in the production of this manuscript.

## Data Availability

The original contributions presented in the study are included in the article/supplementary material. Further inquiries can be directed to the corresponding author.
